# Predictive value of different baseline optical coherence tomography biomarkers for visual acuity changes in neovascular age-related macular degeneration

**DOI:** 10.1186/s40942-025-00633-0

**Published:** 2025-02-05

**Authors:** Hamid Riazi-Esfahani, Hooshang Faghihi, Fatemeh Bazvand, Mohammadreza Mehrabi Bahar, Hassan Khojasteh, Ahmed Husein Ahmed, Shahin Faghihi, Ali Fakhraie, Mohammad Hossein Zamani, Samin Ghasemi, Esmaeil Asadi Khameneh, Elias Khalili Pour

**Affiliations:** 1 Farabi Eye Hospital, Tehran University of Medical Sciences, Qazvin Sq, Tehran, Iran; 2 Bascom Palmer Eye Institute, University of Miami, Miami, FL USA; 3 Retinal Disorders and Ophthalmic Genetics Division, Stein Eye Institute, University of California of Los Angeles, David Geffen School of Medicine at UCLA, Los Angeles, CA USA

**Keywords:** Age-related macular degeneration, Choroidal neovascularization, Anti-vascular endothelial growth factor, Optical coherence tomography

## Abstract

**Background:**

To evaluate baseline optical coherence tomography (OCT) biomarkers in treatment-naïve patients with neovascular age-related macular degeneration (nAMD) and their correlation with visual acuity changes following intravitreal aflibercept injections.

**Methods:**

A retrospective analysis was conducted on treatment-naïve nAMD patients. Baseline OCT biomarkers, including shallow irregular pigment epithelial detachment (SIPED), subretinal hyperreflective material, subretinal fluid, intraretinal fluid (IRF), hyperreflective foci, and subretinal drusenoid deposits, were assessed. Patients received bimonthly aflibercept injections after three loading doses. Visual acuity changes were evaluated at 3 and 12 months. The maximum height and width of the largest pigment epithelial detachment (PED) were also measured.

**Results:**

Among 89 eyes with nAMD, mean best-corrected visual acuity (BCVA) improved by 6 Early Treatment Diabetic Retinopathy Study (ETDRS) letters from baseline to month 3, with sustained improvement through month 12. Baseline IRF was associated with poorer visual acuity improvement at month 12, with patients showing a mean improvement of 1.6 ± 18.2 ETDRS letters versus 11.1 ± 10 ETDRS letters in those without IRF (*P* = 0.002). Multivariable analysis indicated SIPED was linked to lower visual gains at month 3 (*P* = 0.025). The largest PED width correlated significantly with lower BCVA gains at months 3 (*P* = 0.021) and 12 (*P* = 0.043), suggesting its potential as a prognostic factor.

**Conclusion:**

Baseline OCT biomarkers, including SIPED, IRF, and PED width, may predict visual acuity changes in nAMD patients treated with aflibercept, highlighting the need for individualized monitoring.

**Clinical trial number:**

Not applicable.

## Introduction

Neovascular age-related macular degeneration (nAMD) is defined as the presence of macular neovascularization (MNV) often associated with subretinal fluid (SRF) and/or intraretinal fluid (IRF) in a patient with age-related macular degeneration (AMD) [1]. In the presence of retinal hemorrhage, SRF, or IRF, nAMD almost always requires treatment with intravitreal injection of anti-vascular endothelial growth factor (Anti-VEGF) [2]. Despite the widespread use of anti-VEGF therapy, predicting treatment response in patients with nAMD remains a significant challenge.

Optical coherence tomography (OCT) has emerged as a valuable tool for diagnosing and monitoring nAMD, but its ability to predict treatment outcomes is limited [3]. The ability to differentiate between patients who responded favorably to treatment and those who did not respond adequately is important for the clinician to guide the treatment protocol. For patients with an inadequate response to therapy, consideration may be given to switching to another anti-VEGF agent, shortening the follow-up period, and using a fixed regimen or treat-and-extend regimen rather than the Pro re Nata (PRN) regimen [2, 4, 5].

Previous studies have investigated various OCT biomarkers to identify patients who may benefit from early intervention or alternative treatment strategies [3, 6, 7]. However, the optimal combination of OCT biomarkers for predicting visual outcomes in nAMD remains unclear.

This study aims to evaluate a comprehensive set of baseline OCT biomarkers in treatment-naïve patients with nAMD and assess their correlation with visual acuity changes following intravitreal aflibercept injections. By identifying reliable predictors of treatment response, we aim to improve the management of nAMD and optimize patient care.

## Methods

This study was a retrospective analysis of treatment-naïve patients with active nAMD referred to Farabi Eye Hospital between 2019 and 2022. The study adhered to the tenets of the Declaration of Helsinki and was approved by the institutional review board committee of the Tehran University of Medical Sciences (Ethical code: IR.TUMS.FARABIH.REC.1401.031).

All patients had previously undergone slit lamp examination, dilated fundus examination, intraocular pressure measurement, and best-corrected visual acuity (BCVA) measurement using standard Early Treatment Diabetic Retinopathy Study (ETDRS) charts, optical coherence tomography (OCT), and fluorescein angiography (FA). nAMD was defined as the presence of macular neovascularization (MNV) with intraretinal and/or subretinal fluid or hemorrhage observed on fundoscopy, along with typical AMD-related changes such as drusen, in patients aged 50 years or older. The study included patients with one locus of MNV with either type 1 or mixed type 1 and type 2 MNV. In cases of mixed type 1 and type 2, the MNV complex was located within both the choriocapillaris layer and the outer retinal space. In cases where the diagnosis was uncertain, indocyanine green angiography (ICGA), enhanced depth imaging optical coherence tomography (EDI-OCT), and optical coherence tomography angiography (OCTA) had been performed to exclude pachychoroid spectrum diseases and other causes of choroidal neovascularization including polypoidal choroidal vasculopathy and type 3 MNV. Patients with poor image quality, significant media opacity, a history of vitreoretinal surgery, any history of intraocular inflammation, diabetes mellitus, and a refractive error of more than 4 diopters of spherical equivalent were excluded.

Macular OCT scans were acquired using a Spectralis SD-OCT device (Heidelberg Engineering, Germany). All horizontal raster B scans were meticulously evaluated by experienced ophthalmologists to identify relevant OCT biomarkers. Patients initially received a loading dose of three monthly intravitreal injections of aflibercept or its biosimilar, Tyalia (CinnaGen Company, Iran). A previous study demonstrated the non-inferiority of Tyalia to Eylea [8]. Following the loading phase, patients were treated with bimonthly injections for up to 12 months, resulting in a total of seven intravitreal injections. Clinical evaluations, including funduscopy, OCT, and best-corrected visual acuity (BCVA) measurements, were conducted at month 3 and every other month until 12 months.

Two unmasked experienced retina specialists (H.R. and E.K.) assessed various OCT biomarkers, including the maximum height and width of the largest pigment epithelial detachment (PED), shallow irregular pigment epithelial detachment (SIPED), subretinal drusenoid deposit (SDD), pre-choroidal cleft, subretinal hyperreflective material (SHRM), hyperreflective foci (HRF), subretinal fluid (SRF), and intraretinal fluid (IRF). If consensus between the two experts could not be reached, a third retina specialist (E.A.) was consulted to address the discrepancy.

**Fig. 1:**
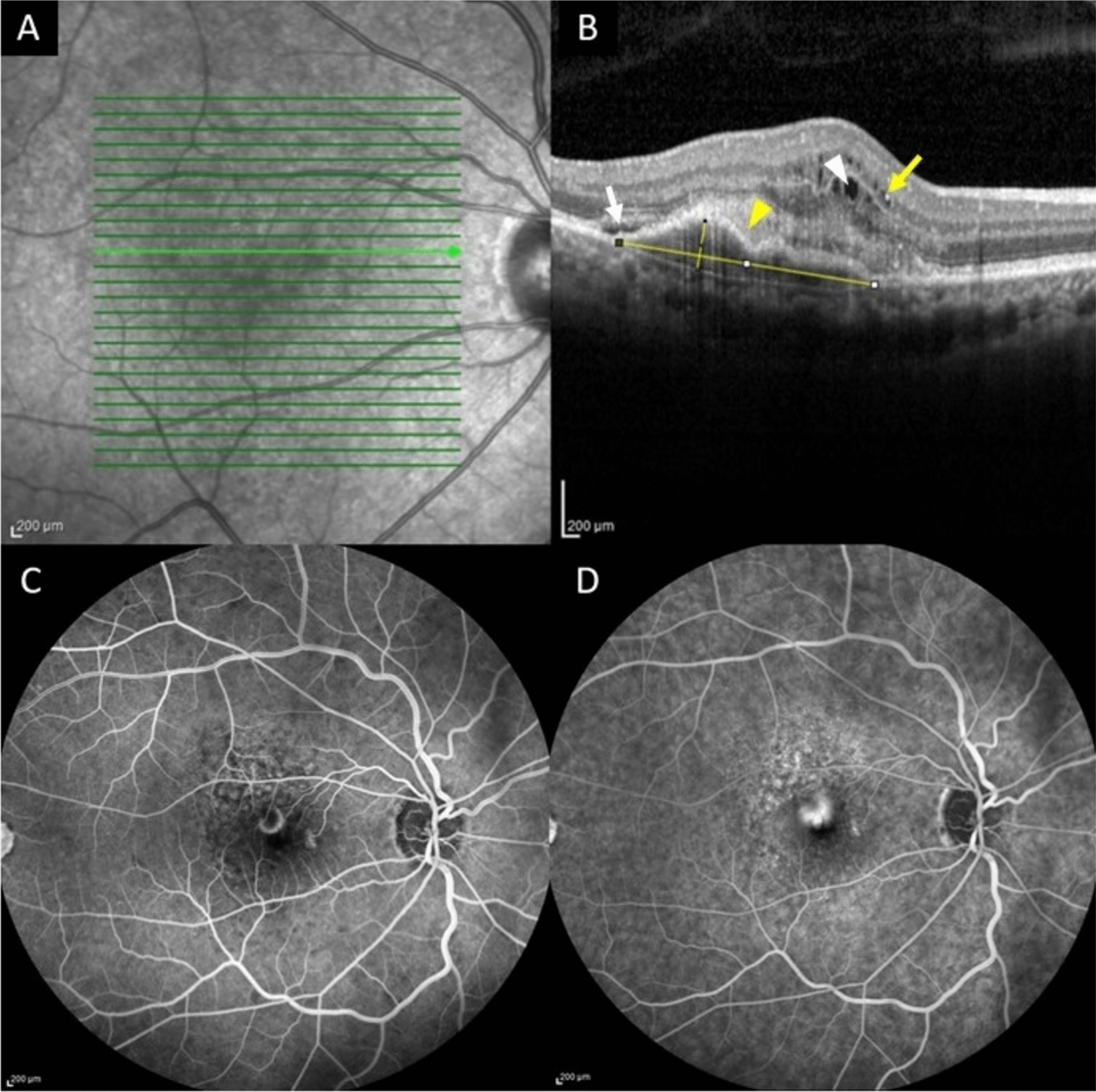
Using calipers, we measured the maximum height and width of the largest pigment epithelial detachment. The *green arrow* on the infrared image (**A**) corresponds to the B-scan optical coherence tomography (**B**), showing the measurement of maximum pigment epithelial detachment height and width (*yellow lines*) using calipers in ImageJ software. The patient also exhibits subretinal fluid (*white arrow*), intraretinal fluid (*white arrowhead*), fibrovascular pigment epithelial detachment (*yellow arrowhead*), and hyperreflective foci (*yellow arrow*). The arteriovenous (**C**) and venous (**D**) phases of fluorescein angiography demonstrate dye leakage consistent with classic choroidal neovascularization

The maximum height and width of the largest pigment epithelial detachment (PED) were meticulously measured using ImageJ software (Rasband, W.S., ImageJ, U.S. National Institutes of Health, Bethesda, Maryland, USA, https://imagej.nih.gov/ij). Prior to measurement, the software’s scale was carefully calibrated using a reference OCT image with a known scale to ensure accurate quantification. The PED width was defined as the horizontal distance between the points where the retinal pigment epithelium (RPE) detaches from Bruch’s membrane (BM) on either side of the largest PED, measured with calipers within the ImageJ software. The PED height was measured as the vertical distance from BM to the base of the detached RPE layer, perpendicular to the line drawn for the width measurement. All raster scans were thoroughly reviewed to identify the maximum PED height and width, which were not necessarily measured on the same raster scan. This approach is visually illustrated in Fig. 1.

**Fig. 2:**
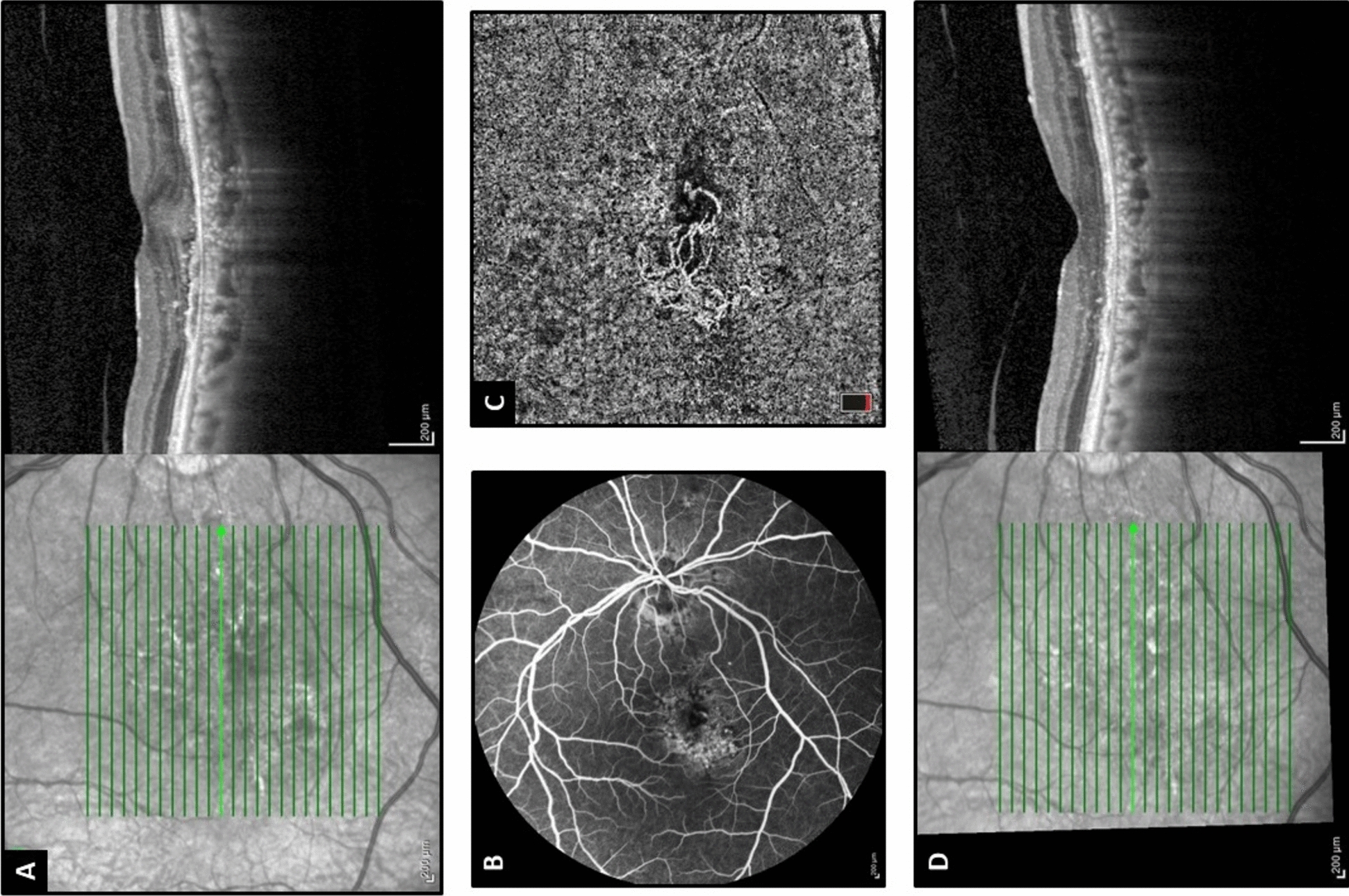
A patient with neovascular age-related macular degeneration. **A** Optical coherence tomography (OCT) image shows a shallow irregular pigment epithelial detachment (SIPED) with overlying subretinal fluid and hyperreflective foci. **B** Fluorescein angiography demonstrates stippled fluorescein leakage in the central macular area. **C** The choriocapillaris slab of OCT angiography reveals a macular neovascular complex. **D** OCT at the same location after one year of treatment with intravitreal aflibercept injections shows resolution of subretinal fluid

SIPED was defined as a shallow and irregular elevation of the hyperreflective RPE layer from the hyperreflective BM beneath it, with a minimum horizontal and maximum vertical dimension of 500 and 100 microns, respectively [9–11] (Fig. 2). SDD was defined as at least three hyperreflective or moderately reflective (in comparison to RPE reflectivity) cones or small mound between the ellipsoid zone and RPE [12]. Pre-choroidal cleft was defined as a hypo reflective space between the RPE and BM associated with posterior bowing of BM [6, 13]. HRF were defined as focal, well-circumscribed lesions with reflectivity greater than that of the RPE layer, qualitatively assessed and distinguishable from retinal vessel cross-sections, without any limitation on their number [14, 15]. The presence or absence of SHRM, intraretinal, or subretinal HRF, SRF, and IRF were also evaluated in the OCT scan of each patient.

The change in BCVA from baseline to month 3 and from baseline to month 12 for each patient was calculated. The patients were divided into two groups based on the changes in BCVA from baseline to month 12. Patients with BCVA improvement equal to or greater than 15 ETDRS letters were defined as the improvement group, and patients with BCVA worsening equal to or greater than 15 ETDRS letters were defined as the worsening group. This grouping was conducted to evaluates the baseline anatomical biomarkers that could potentially impact visual acuity changes with clinically greater magnitudes.

The primary outcome of the study was the change in BCVA from baseline to months 3 and 12, as measured in ETDRS letters.

To achieve 80% power to detect a correlation of at least 0.3 between OCT parameters and changes in BCVA, a minimum sample size of 84 participants was calculated, assuming a type I error rate of 0.05.

To present data, we utilized mean, standard deviation, median, frequency, and percentage. When comparing variables between two groups at different follow-up times, considering the potential correlation of outcomes in bilateral eyes, we employed generalized estimating equation (GEE) analysis. To assess the simultaneous effect of variables on visual acuity (VA) over the study period, we examined the interaction of time and variables in a multivariable GEE analysis. All variables with a *P*-value < 0.100 in simple GEE analysis at any time point (3 or 12 months of follow-up) were included in this analysis. All statistical analyses were performed using SPSS (IBM Corp. Released 2017. IBM SPSS Statistics for Windows, Version 25.0. Armonk, NY: IBM Corp). A *P*-value < 0.05 was considered statistically significant.

## Results

In this study, 89 eyes with nAMD from 88 patients were included. The mean age of the patients was 68.8 ± 6.4 years, with a median age of 69 years (range: 55–80 years). Among them, 57 (64%) patients were male, and 32 (36%) patients were female. MNV was detected in the right eye in 46 (51.7%) patients and the left eye in 43 (48.3%) patients. The mean BCVA of the patients at baseline, month 3, and month 12 were 54 ± 12, 60 ± 15, and 60 ± 19 ETDRS letters, respectively. The mean change in BCVA from baseline to month 3 and from baseline to month 12 was 6 ± 12 and 6 ± 16 ETDRS letters, respectively.

At baseline, SHRM, SRF, subretinal HRF, intraretinal HRF, and SDD were present in 84.3, 83.1, 77.5, 75.3, and 18% of patients, respectively. However, none of these biomarkers were significantly associated with changes in BCVA over time (Table 1).

**Table 1:** The association between baseline optical coherence tomography markers of patients and the change in BCVA from baseline to months 3 and 12

Variable	Number (%)	BCVA change in month 3 value			BCVA change in month 12 value			Multivariable^‡^
		Mean ± SD	Median	*P*^†^	Mean ± SD	Median	*P*^†^	
SIPED								
No	33 (37.1%)	9.4 ± 13.5	8	0.051	8.5 ± 17.8	12	0.189	**0.025**
Yes	56 (62.9%)	4 ± 10.6	5.5		3.7 ± 14.6	5.5		
SHRM								
No	14 (15.7%)	1.8 ± 9.8	1.5	0.1	5.3 ± 11	7.5	0.92	–
Yes	75 (84.3%)	6.8 ± 12.2	7		5.6 ± 16.8	7.5		
SRF								
No	15 (16.9%)	0.7 ± 11.9	−1	0.065	−0.3 ± 19.8	5	0.211	0.856
Yes	74 (83.1%)	7.1 ± 11.8	7		6.7 ± 15.1	8		
IRF								
No	36 (40.4%)	8.3 ± 9	7	0.124	11.1 ± 10	10.5	**0.002**	**0.021**
Yes	53 (59.6%)	4.5 ± 13.6	5.5		1.6 ± 18.2	3.5		
HRF (subretinal)								
No	20 (22.5%)	2.7 ± 12.1	2	0.125	1.7 ± 19.5	7	0.309	–
Yes	69 (77.5%)	7 ± 11.9	7		6.6 ± 14.9	7.5		
HRF (intraretinal)								
No	22 (24.7%)	6.8 ± 8	7.5	0.688	8.2 ± 9.2	6.5	0.252	–
Yes	67 (75.3%)	5.8 ± 13.1	5.5		4.7 ± 17.6	8		
SDD								
No	73 (82.0%)	6.9 ± 11.3	7	0.234	6.7 ± 16.1	9	0.172	–
Yes	16 (18.0%)	2.5 ± 14.3	5.5		1 ± 15.1	3		
Pre-choroidal cleft								
No	84 (94.4%)	5.6 ± 11.9	6	**0.027**	5.2 ± 16.1	7	0.184	**0.01**
Yes	5 (5.6%)	18 ± 11.5	19		14.3 ± 14	13		

^†^Based on GEE analysis

^‡^Based on interaction of Time and variable in a multivariable GEE analysis. All variables which have a *P*-value < 0.100 in simple GEE analysis at any time (3 or 12 months) entered into this analysis. Bold values indicate statistically significant differences

*BCVA* best-corrected visual acuity, *SIPED* shallow irregular pigment epithelial detachment, *SHRM* subretinal hyperreflective material, *SRF* subretinal fluid, *IRF* intraretinal fluid, *HRF* hyperreflective foci, *SDD* subretinal drusenoid deposit

**Fig. 3:**
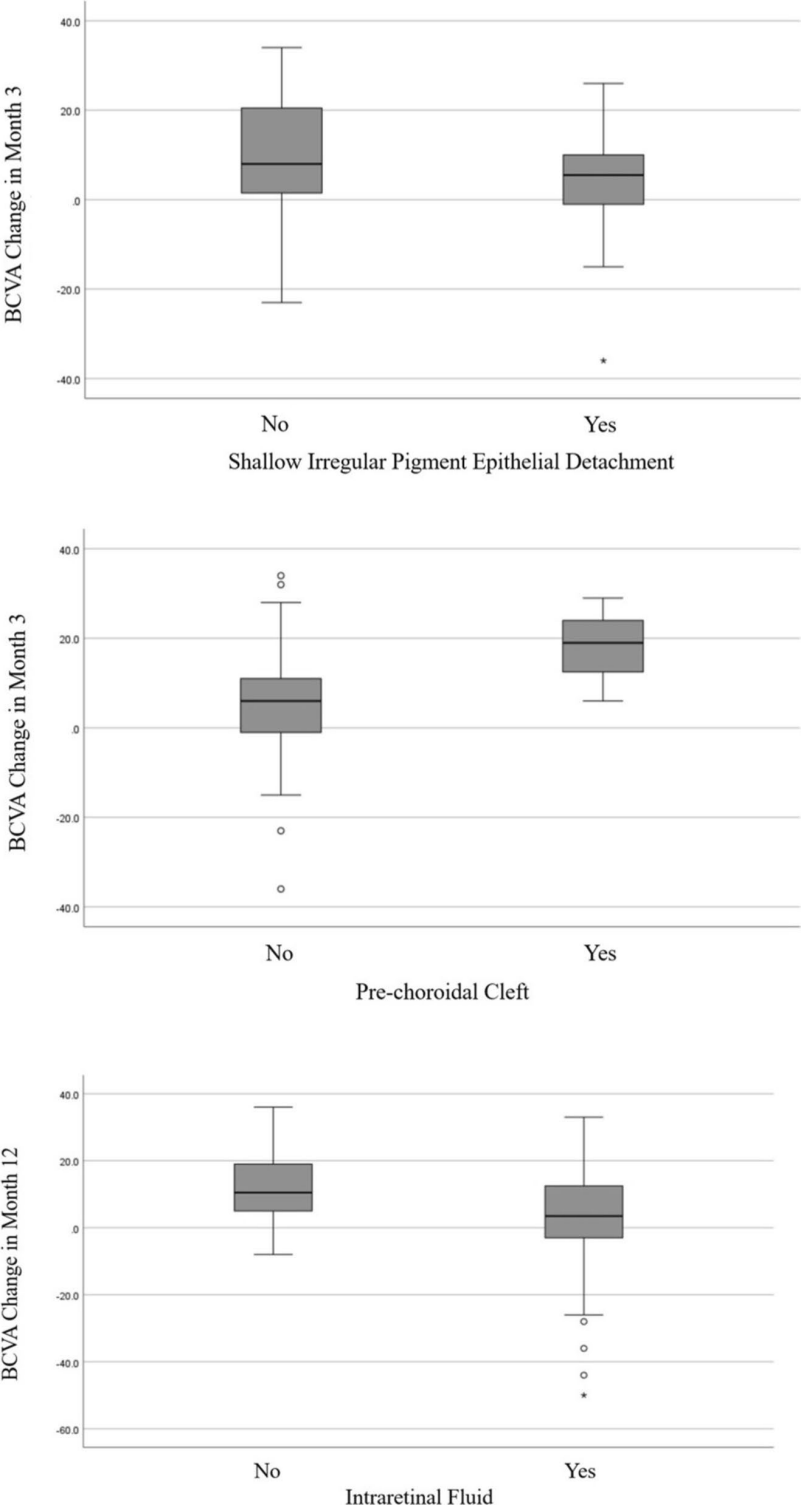
Changes in best-corrected visual acuity (BCVA) measured in Early Treatment Diabetic Retinopathy Study (ETDRS) letters across different patient groups. *Top*: Boxplot comparing the change in BCVA from baseline to month 3 in patients with and without shallow irregular pigment epithelial detachment. *Middle*: Boxplot comparing the change in BCVA from baseline to month 3 in patients with and without pre-choroidal cleft. *Bottom*: Boxplot comparing the change in BCVA from baseline to month 12 in patients with and without intraretinal fluid

Shallow irregular pigment epithelial detachment (SIPED) was present in 62.9% of patients. While univariate analysis did not reveal a significant association between SIPED and changes in visual acuity, multivariable analysis demonstrated that patients with SIPED experienced lower visual gains at month 3 compared to those without SIPED (4 ± 10.6 vs. 9.4 ± 13.5 ETDRS letters, *P* = 0.025). IRF was present in 59.6% of patients at baseline, and these patients showed a lower gain in BCVA from baseline to month 12 compared to patients without IRF (1.6 ± 18.2 compared to 11.1 ± 10 ETDRS letters) (Fig. 3). This difference was statistically significant in both univariate and multivariate analyses (*P* = 0.002 and 0.021, respectively).

**Fig. 4:**
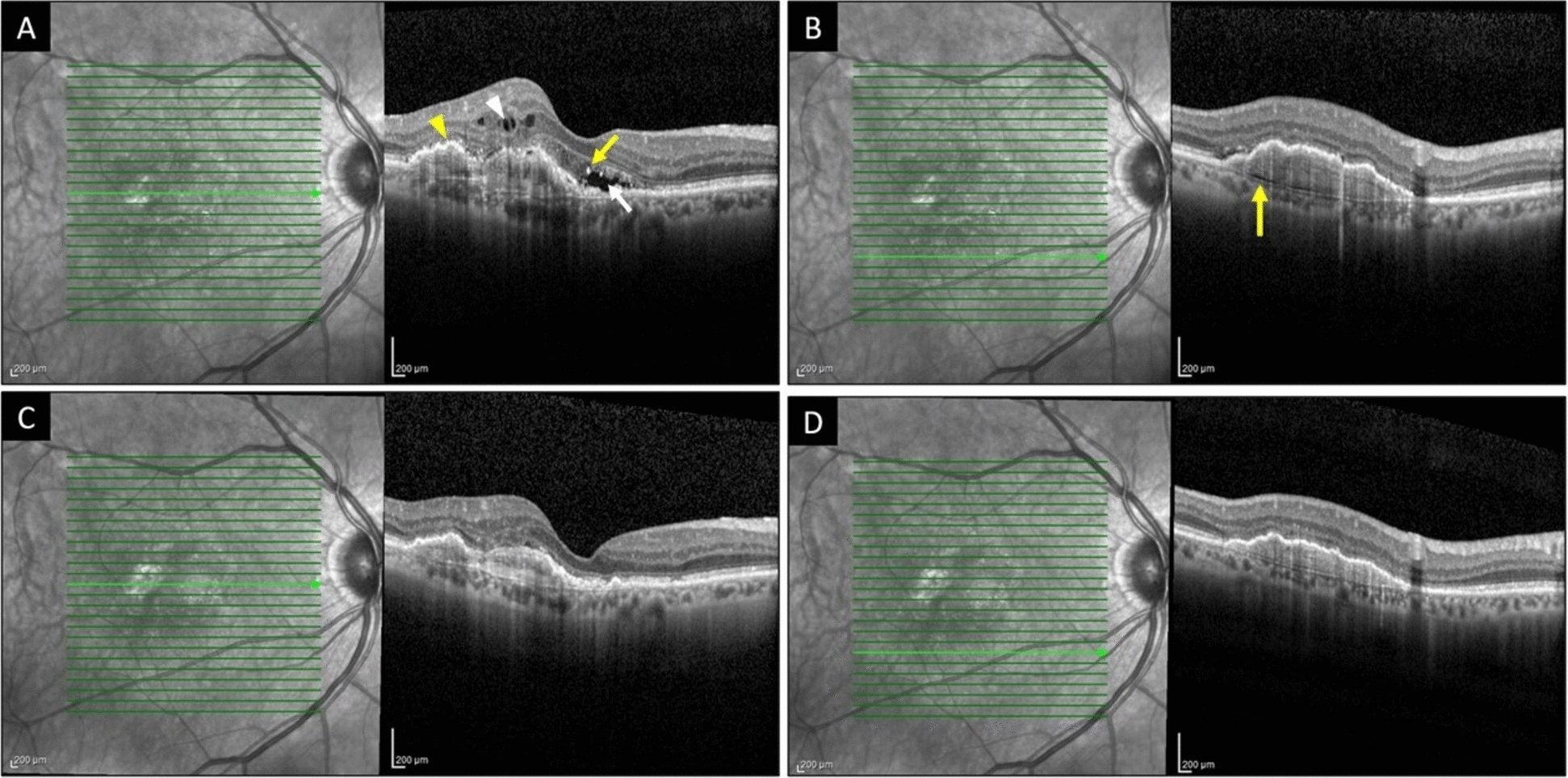
A patient with neovascular age-related macular degeneration. Optical coherence tomography at baseline (**A**, **B**) demonstrates fibrovascular pigment epithelial detachment (*yellow arrowhead*), intraretinal fluid (*white arrowhead*), subretinal fluid (*white arrow*), hyperreflective foci (*yellow arrow* in **A**), and a pre-choroidal cleft (*yellow arrow* in **B**). The corresponding optical coherence tomography after 12 months of intravitreal aflibercept treatment (**C**, **D**) shows a dry macula with resolution of fluid compartments

Pre-choroidal cleft was present in 5.6% of patients and was significantly associated with a greater gain in BCVA from baseline to month 3 in both univariate and multivariate analyses (*P* = 0.027 and 0.01, respectively). However, this association was not observed when considering the change in BCVA from baseline to month 12 (*P* = 0.184). Figure 4 shows a representative case of a patient with a pre-choroidal cleft at baseline.

**Fig. 5:**
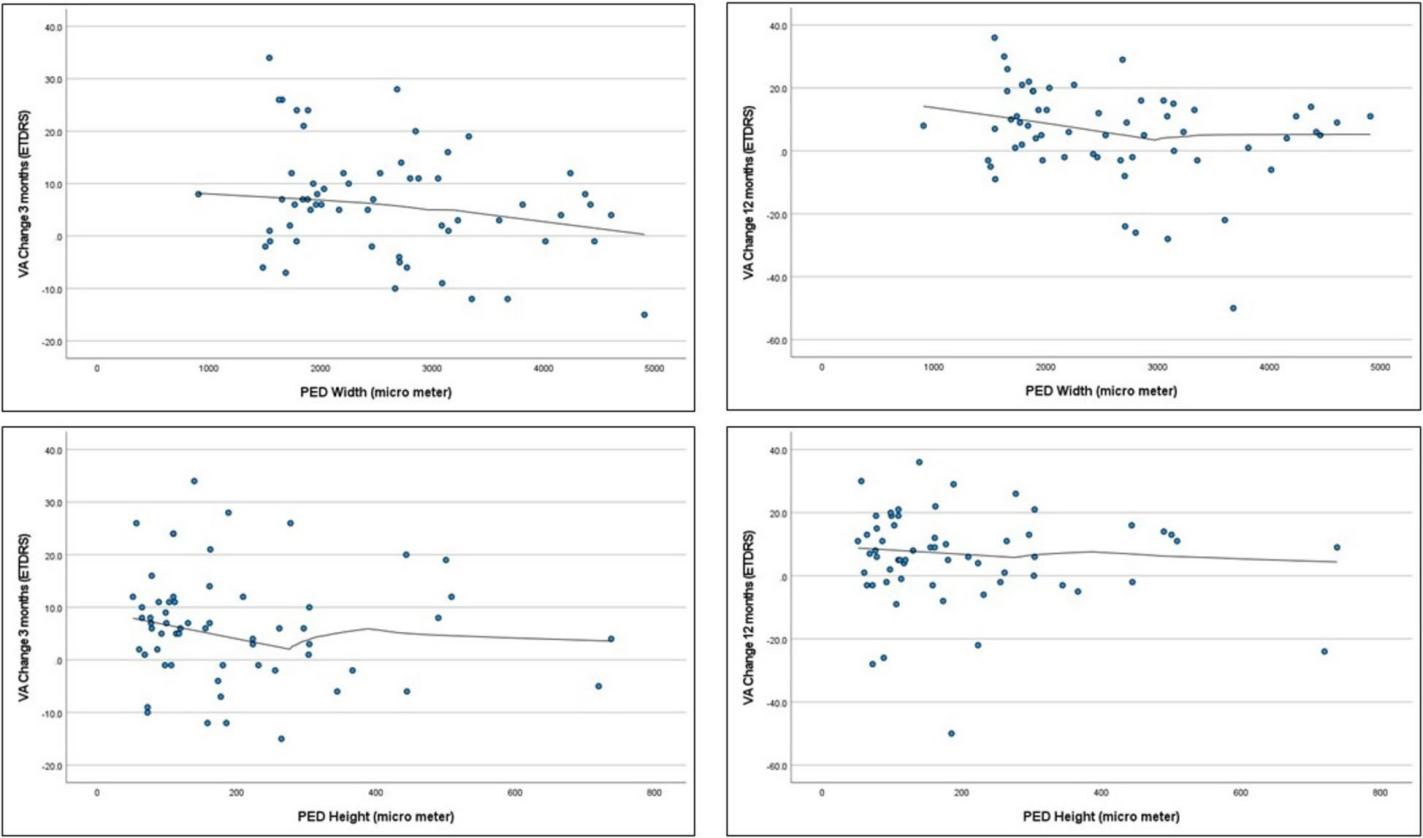
Correlation of pigment epithelial detachment (PED) dimensions with best-corrected visual acuity (BCVA) changes at various time points. A larger PED width at baseline was significantly associated with smaller visual acuity gains at month 3 (*top left*) and month 12 (*top right*). In contrast, PED height showed no significant association with visual acuity changes at month 3 (*bottom left*) or month 12 (*bottom right*)

The mean of the largest PED height and width at baseline were 197 ± 151 (median 154) and 2708 ± 1058 (median 2602) micrometers, respectively. The mean largest PED height was not associated with BCVA changes at either 3 months or 12 months from baseline (*P* = 0.335 and 0.546, respectively). However, the mean largest PED width was significantly associated with lower BCVA gains at both 3 months and 12 months from baseline. (Fig. 5) The generalized linear model indicates that each micrometer increase in the largest PED width was associated with a decrease of 0.003 ± 0.0013 (95% confidence interval: −0.006 to 0.0001, *P* = 0.021) and 0.004 ± 0.0019 (95% confidence interval: −0.007 to 0.0001, *P* = 0.043) ETDRS letters from baseline to month 3 and month 12, respectively.

Among 14 patients with vision improvement of more than 15 ETDRS letters from baseline to month 3, the absence of SIPED at baseline was significantly related to vision improvement (*P* = 0.04). There were 22 patients with vision improvement of more than 15 ETDRS letters from baseline to month 12. The only statistically significant baseline OCT biomarker related to vision improvement of more than 15 ETDRS letters from baseline to month 12 was also the absence of SIPED (*P* = 0.027) (Table 2).

**Table 2:** The baseline optical coherence tomography markers of patients with vision improvement of more than 15 ETDRS letters from baseline to months 3 and 12

Variable	Vision gain in month 3			Vision gain in month 12		
	15 ≥ ETDRS letters *N* (%)	15 < ETDRS letters *N* (%)	*P*^†^	15 ≥ ETDRS letters *N* (%)	15 < ETDRS letters *N* (%)	*P*^†^
SIPED						
No	23 (71.9%)	9 (28.1%)	**0.04**	19 (59.4%)	13 (40.6%)	**0.027**
Yes	45 (90.0%)	5 (10.0%)		41 (82.0%)	9 (18.0%)	
SHRM						
No	11 (91.7%)	1 (8.3%)	0.398	10 (83.3%)	2 (16.7%)	0.397
Yes	57 (81.4%)	13 (18.6%)		50 (71.4%)	20 (28.6%)	
SRF						
No	11 (84.6%)	2 (15.4%)	0.86	11 (84.6%)	2 (15.4%)	0.32
Yes	57 (82.6%)	12 (17.4%)		49(71.0%)	20 (29.0%)	
IRF						
No	28 (82.4%)	6 (17.6%)	0.907	22 (64.7%)	12 (35.3%)	0.149
Yes	40 (83.3%)	8 (16.7%)		38 (79.2%)	10 (20.8%)	
HRF (subretinal)						
No	15 (83.3%)	3 (16.7%)	0.969	14 (77.8%)	4 (22.2%)	0.618
Yes	53 (82.8%)	11 (17.2%)		46 (71.9%)	18 (28.1%)	
HRF (intraretinal)						
No	18 (90.0%)	2 (10.0%)	0.643	15 (75.0%)	5 (25.0%)	0.842
Yes	50 (80.6%)	12 (19.4%)		45 (72.6%)	17 (27.4%)	
SDD						
No	54 (81.8%)	12 (18.2%)	0.59	45 (68.2%)	21 (31.8%)	0.068
Yes	14 (87.5%)	2 (12.5%)		15 (93.8%)	1 (6.3%)	
Pre-choroidal cleft						
No	67 (84.8%)	12 (15.2%)	0.056	58 (73.4%)	21 (26.6%)	0.796
Yes	1 (33.3%)	2 (66.7%)		2 (66.7%)	1 (33.3%)	

^†^Based on GEE analysis. Bold values indicate statistically significant differences

*BCVA* best-corrected visual acuity, *SIPED* shallow irregular pigment epithelial detachment, *SHRM* subretinal hyperreflective material, *SRF* subretinal fluid, *IRF* intraretinal fluid, *HRF* hyperreflective foci, *SDD* subretinal drusenoid deposit

There were 3 and 7 patients with vision loss of more than 15 ETDRS letters from baseline to months 3 and 12, respectively. In these two subgroups, IRF was present in all patients and it was the only statistically significant baseline OCT biomarker related to vision loss of more than 15 ETDRS letters from baseline to month 12 (*P* = 0.038) (Table 3).

**Table 3:** The baseline optical coherence tomography markers of patients with vision loss of more than 15 ETDRS letters from baseline to months 3 and 12

	Vision loss in month 3			Vision loss in month 12		
	15 ≥ ETDRS letters *N* (%)	15 < ETDRS letters *N* (%)	*P*^†^	15 ≥ ETDRS letters *N* (%)	15 < ETDRS letters *N* (%)	*P*^†^
SIPED						
No	31 (96.9%)	1 (3.1%)	0.837	29 (90.6%)	3 (9.4%)	0.828
Yes	48 (96.0%)	2 (4.0%)		46 (92.0%)	4 (8.0%)	
SHRM						
No	11 (91.7%)	1 (8.3%)	0.382	12 (100.0%)	0 (0.0%)	0.586
Yes	68 (97.1%)	2 (2.9%)		63 (90.0%)	7 (10.0%)	
SRF						
No	12 (92.3%)	1 (7.7%)	0.408	11 (84.6%)	2 (15.4%)	0.306
Yes	67 (97.1%)	2 (2.9%)		64 (92.8%)	5 (7.2%)	
IRF						
No	34 (100.0%)	0 (0.0%)	0.263	34 (100.0%)	0 (0.0%)	**0.038**
Yes	45 (93.8%)	3 (6.3%)		41 (85.4%)	7 (14.6%)	
HRF (subretinal)						
No	17 (94.4%)	1 (5.6%)	0.53	15 (83.3%)	3 (16.7%)	0.175
Yes	62 (96.9%)	2 (3.1%)		60 (93.8%)	4 (6.3%)	
HRF (intraretinal)						
No	20 (100.0%)	0 (0.0%)	0.573	20 (100.0%)	0 (0.0%)	0.186
Yes	59 (95.2%)	3 (4.8%)		55 (88.7%)	7 (11.3%)	
SDD						
No	64 (97.0%)	2 (3.0%)	0.483	61 (92.4%)	5 (7.6%)	0.618
Yes	15 (93.8%)	1 (6.3%)		14 (87.5%)	2 (12.5%)	
Pre-choroidal cleft						
No	76 (96.2%)	3 (3.8%)	>0.99	72 (91.1%)	7 (8.9%)	>0.99
Yes	3 (100.0%)	0 (0.0%)		3 (100.0%)	0 (0.0%)	

^†^Based on GEE analysis. Bold values indicate statistically significant differences

*BCVA* best-corrected visual acuity, *SIPED* shallow irregular pigment epithelial detachment, *SHRM* subretinal hyperreflective material, *SRF* subretinal fluid, *IRF* intraretinal fluid, *HRF* hyperreflective foci, *SDD* subretinal drusenoid deposit

## Discussion

Neovascular AMD remains a significant cause of vision impairment and blindness in the elderly population [1]. Despite advancements in treatment modalities, including anti-VEGF therapy, individual responses to treatment vary, posing challenges for clinicians in predicting visual outcomes and optimizing management strategies.

Our study evaluated various OCT biomarkers to determine their association with changes in visual acuity after treatment. We found that the presence of specific features such as SIPED, IRF, pre-choroidal cleft, and the largest PED width demonstrated significant associations with changes in visual acuity at different time points during the study period. Conversely, other OCT biomarkers, including SHRM, SRF, HRF, SDD, and the largest PED height, did not show significant associations with changes in visual acuity during the first year of intravitreal anti-VEGF therapy.

In the present study, patients with SIPED exhibited significantly lower visual gains at month 3 compared to those without SIPED in multivariable analyses. Additionally, they exhibited significantly lower visual gains of more than 15 ETDRS letters at both month 3 and month 12, with a strong association with overall changes in visual acuity. Few studies have evaluated the prognostic value of SIPED in patients with nAMD, and the results have been mixed. Some researchers have hypothesized that SIPED may be a morphological marker of polypoidal choroidal vasculopathy (PCV), a condition often associated with less favorable responses to anti-VEGF therapy compared to photodynamic therapy [16]. It is suggested that patients with nAMD and SIPED may similarly have a less satisfactory response to anti-VEGF therapy alone [17]. Additionally, differences in the morphology of neovascular complexes between AMD patients with and without SIPED have been reported. In patients with nAMD and SIPED, the vessel area, total vessel length, and average vessel length in the MNV complex are greater compared to patients with nAMD without SIPED. These morphological variations may influence the degree of response to anti-VEGF treatment, further supporting the potential prognostic significance of SIPED [18].

The presence of IRF was consistently associated with poorer vision gains, underscoring its detrimental effect on treatment response. We found that the presence of IRF was associated with vision loss of more than 15 ETDRS letters from baseline to both month 3 and month 12. These findings align with previous studies suggesting that the presence of IRF may indicate a more aggressive disease phenotype associated with resistance to anti-VEGF therapy [19–23]. The presence of IRF may indicate that the RPE and neural retina have sustained more severe damage compared to the presence of SRF. This severe damage can lead to fluid accumulation within the retinal tissue due to compromised retinal integrity, potentially resulting in irreversible damage [24].

In contrast, our study, along with others, indicates that the presence of SRF is not consistently linked to poorer visual gains. Evidence indicates that SRF observed at various time points during treatment and follow-up, without significant fluctuations, does not necessarily correlate with worse visual results. However, fluctuations in SRF levels may negatively impact visual outcomes [24–26].

We did not find a significant association between the presence of SHRM and reduced gains in visual acuity. This finding contrasts with other research that has reported a negative correlation between SHRM and visual response. Multiple studies suggest that SHRM may adversely impact visual outcomes in patients with nAMD [22, 27]. Despite this, our results imply that SHRM alone may not serve as a definitive predictor of poor visual response. It’s worth noting that the presence of SHRM in nearly 85% of the patients may have weakened the credibility of this analysis.

In our study, a pre-choroidal cleft was present in 5.6% of patients and was associated with BCVA gain from baseline to month 3; however, this gain was not observed from baseline to month 12. Unlike our findings, most similar studies have shown that the presence of a pre-choroidal cleft is associated with a poor visual prognosis. The prechoroidal cleft has recently been identified as a distinct OCT feature in nAMD, and there are currently conflicting theories regarding its clinical significance. Earlier studies suggested a potential link between the cleft and MNV activity others have interpreted the cleft as a potential chronic structural change associated with the neovascular tissue [28]. A study by Hayashi-Mercado et al. evaluated OCT biomarkers as prognostic factors in nAMD treatment. A total of 83 patients (18 presenting with a pre-choroidal cleft) were evaluated, and BCVA gain after one month of treatment was significantly higher in the absence of a pre-choroidal cleft (+3.7 ETDRS letters, *P* = 0.04), revealing that a pre-choroidal cleft is associated with lower visual gain [29]. In a study by Kredi et al., a pre-choroidal cleft was found in 15% of 140 nAMD patients. After treatment with intravitreal anti-VEGF and a minimum follow-up duration of 24 months, BCVA improved significantly (*P* = 0.008); however, the change in BCVA was not related to the presence of a pre-choroidal cleft (*P* = 0.208) [30]. Nonetheless, a significant correlation was found between the presence of a pre-choroidal cleft and central subfield thickness, PED height, multi-layered PED, and having SRF over the follow-up period, concluding that a pre-choroidal cleft correlates with disease activity and a need for more anti-VEGF injections [30]. In another study by Kim Jong Min et al., 234 patients treated for nAMD were assessed, and visual acuity was compared between patients manifesting a pre-choroidal cleft and a control group after at least 12 months of follow-up. 8.1% of the patients had a pre-choroidal cleft, and visual acuity in the pre-choroidal cleft group was significantly worse than in the control group (*P* = 0.049). This study concluded that eyes with a cleft, especially those that developed early (within 6 months of treatment initiation), had a worse prognosis compared to the control group [31]. The cleft might represent a fluid pocket accumulated between the fibrovascular tissue and BM. Similar to SRF, the fluid forming the prechoroidal cleft could respond variably to anti-VEGF treatment, but its fluctuations appear to be related to MNV exudation, potentially serving as an additional indicator of disease activity. Therefore, the presence of a prechoroidal cleft at baseline that remains stable during anti-VEGF therapy may not necessarily affect vision [6, 32]. The total number of patients with pre-choroidal cleft in our study is relatively small. The difference in visual outcome findings between our study and the mentioned studies could be attributed to the limited sample size of our study, follow-up time, and the distribution of MNV subtypes.

The height and width of PED and their association with BCVA changes were measured in our study. Although the mean largest PED height was not significantly associated with BCVA changes, the mean largest PED width showed a significant association with BCVA changes. Specifically, each micrometer increase in the largest PED width was associated with a decrease of 0.003 and 0.004 ETDRS letters from baseline to month 3 and month 12, respectively. Many studies have established the association between the presence of PED at baseline and visual outcomes after treatment; however, only a few studies have analyzed specific morphological parameters of PED as prognostic factors [33–42].

In a study by Sarraf et al., PED thickness was measured at baseline and after 96 weeks of anti-VEGF therapy in 1459 patients [43]. Greater PED thickness was associated with both neovascular activity and lower BCVA gain. Specifically, eyes with PED > 200 µm gained 4.6 letters compared to 7 letters in eyes with PED < 200 µm. Eyes with PED > 300 µm gained 4 letters after treatment, while those with PED < 300 µm gained 6.6 letters. Similarly, eyes with PED > 400 µm gained 3 letters compared to 6.5 letters in eyes with PED < 400 µm [43]. Yiyang Shu et al. studied the correlation of PED morphology at baseline and after 3 and 12 months of anti-VEGF therapy in patients with nAMD. In 82 eyes with nAMD that did not have polypoidal lesions, BCVA gain after both 3 and 12 months of treatment was associated with PED volume (*P* = 0.027, 0.037), and BCVA gain after 12 months was associated with PED width (*P* = 0.044). They concluded that a wider PED width at baseline correlates with less BCVA gain at 12 months in nAMD, suggesting the potential use of these measurements as predictive tools in the treatment of nAMD in the future [44].

Several studies have found PED measurements to be irrelevant to BCVA gains. For instance, Tyagi et al. recruited 50 eyes receiving anti-VEGF therapy, measuring PED height and width at different time points during treatment. Injections began with ranibizumab and were switched to aflibercept, with measurements taken 4 months and 1 year after therapy. BCVA was not associated with any PED measurements after one year of treatment [45]. In a study by Kai Xiong Cheong et al., morphological parameters of PED (width, height, volume, and shape) were compared in 40 patients with nAMD and 38 patients with PCV after 1 year of anti-VEGF treatment. None of the morphological parameters of PED were significantly associated with BCVA changes in the nAMD group [46].

Although we did not find any association between changes in visual acuity at months 3 and 12 and the presence of SDD at baseline, some studies suggest that baseline SDD may be associated with worse visual and anatomical outcomes in nAMD patients treated with anti-VEGF agents [47, 48]. These studies propose that poorer visual gains in such patients could be attributed to thinner subfoveal choroidal thickness and a higher likelihood of macular atrophy development [47]. However, other studies have reported no significant association between the presence of baseline SDD and visual outcomes after treatment in nAMD patients [49, 50].

Overall, our findings contribute to the growing body of evidence supporting the utility of baseline OCT biomarkers in predicting treatment outcomes in nAMD patients. By identifying patients at higher risk of poor response to treatment, clinicians can tailor management strategies accordingly, optimizing visual outcomes and improving patient care.

Limitations of our study include its retrospective nature and relatively small sample size. Additionally, longer-term follow-up and assessment of additional OCT biomarkers, considering changes in these biomarkers over time, may provide further insights into disease progression and treatment response. There is also a lack of OCTA imaging for all patients to differentiate between those with type 1 MNV and those with mixed type 1 and type 2 MNV, as the prognosis and treatment response may differ between these two groups. Moreover, this research was conducted at a tertiary care facility, which may have resulted in the recruitment of a disproportionate number of chronic cases, potentially introducing selection bias.

In conclusion, our study highlights the significance of baseline OCT biomarkers like IRF, SIPED, and wide PED in predicting visual gains in treatment-naïve patients with nAMD. Further prospective studies are warranted to validate these findings and explore additional predictors of treatment response, ultimately enhancing the management of this sight-threatening condition.

## Data Availability

The data supporting the findings of this study are not openly available due to sensitivity concerns but can be obtained from the corresponding author upon reasonable request. The data are stored in controlled-access storage at Farabi Eye Hospital, Tehran University of Medical Sciences.
